# Estratégias organizacionais no centro cirúrgico diante da pandemia de COVID 19: uma revisáo integrativa*

**DOI:** 10.15649/cuidarte.2323

**Published:** 2022-10-17

**Authors:** José Erivelton de Souza Maciel Ferreira, Lidia Rocha de Oliveira, Karoline Galvao Pereira, Natasha Marques Frota, Tahissa Frota Cavalcante, Alana Santos Monte, Anne Fayma Lopes Chaves

**Affiliations:** 1 Universidade da Integrado Internacional da Lusofonia Afro-Brasileira, Redencao (CE), Brasil. Email: eriveltonsmf@gmail.com Universidade da Integração Internacional da Lusofonia Afro-Brasileira Universidade da Integrado Internacional da Lusofonia Afro-Brasileira Redencao CE Brazil eriveltonsmf@gmail.com; 2 Universidade da Integrado Internacional da Lusofonia Afro-Brasileira, Redencao (CE), Brasil. Email: lidiarocha2021@gmail.com Universidade da Integração Internacional da Lusofonia Afro-Brasileira Universidade da Integrado Internacional da Lusofonia Afro-Brasileira Redencao CE Brazil lidiarocha2021@gmail.com; 3 Universidade da Integrado Internacional da Lusofonia Afro-Brasileira, Redencao (CE), Brasil. Email: karoline galvaojk@hotmail.com Universidade da Integração Internacional da Lusofonia Afro-Brasileira Universidade da Integrado Internacional da Lusofonia Afro-Brasileira Redencao CE Brazil karoline galvaojk@hotmail.com; 4 Universidade da Integrado Internacional da Lusofonia Afro-Brasileira, Redencao (CE), Brasil. Email: natasha@unilab.edu.br Universidade da Integração Internacional da Lusofonia Afro-Brasileira Universidade da Integrado Internacional da Lusofonia Afro-Brasileira Redencao CE Brazil natasha@unilab.edu.br; 5 Universidade da Integrado Internacional da Lusofonia Afro-Brasileira, Redencao (CE), Brasil. Email: tahissa@unilab.edu.br Universidade da Integração Internacional da Lusofonia Afro-Brasileira Universidade da Integrado Internacional da Lusofonia Afro-Brasileira Redencao CE Brazil tahissa@unilab.edu.br; 6 Universidade da Integrado Internacional da Lusofonia Afro-Brasileira, Redencao (CE), Brasil. Email: alanamonte@unilab.edu.br Universidade da Integração Internacional da Lusofonia Afro-Brasileira Universidade da Integrado Internacional da Lusofonia Afro-Brasileira Redencao CE Brazil alanamonte@unilab.edu.br; 7 Universidade da Integrado Internacional da Lusofonia Afro-Brasileira, Redencao (CE), Brasil. Email: annefayma@unilab.edu.br Universidade da Integração Internacional da Lusofonia Afro-Brasileira Universidade da Integrado Internacional da Lusofonia Afro-Brasileira Redencao CE Brazil annefayma@unilab.edu.br

**Keywords:** Infeccóes por Coronavirus, Centros Cirúrgicos, Procedimentos Cirúrgicos Operatórios, Coronavirus Infections, Surgicenters, Surgical Procedures, Operative, Infecciones por Coronavirus, Centros Quirúrgicos, Procedimientos Quirúrgicos Operativos

## Abstract

**Introdujo:**

os sistemas de saúde foram desafiados a desenvolver estratégias organizacionais para a prestacao de cuidados cirúrgicos.

**Objetivo:**

apresentar as estratégias dos servidos de saúde no que se refere as práticas de cuidados cirúrgicos em tempos de pandemia de COVID-19.

**Materiais e métodos:**

revisao integrativa, desenvolvida em seis etapas, cuja busca dos artigos ocorreu na Biblioteca Virtual de Saúde, SciELO, PubMed e ScienceDirect. Os descritores controlados em saúde adotados foram “Centros Cirúrgicos” e "Infeccóes por Coronavirus” de acordo com os sistemas DeCS e MeSH Terms. Foram selecionados 60 artigos de 405 estudos encontrados.

**Resultados:**

as principais estratégias utilizadas pelos servidos de saúde foram: a suspensao e adiamento de cirurgias eletivas durante as ondas de contágio da doenca; e a triagem cuidadosa dos pacientes para COVID-19 antes e após intervencóes cirúrgicas.

**Discussáo:**

a suspensao e o adiamento de cirurgias eletivas devem ser avaliados com cautela pela equipe de saúde, de forma individualizada, para cada paciente, visto que situacóes clinicas nao urgentes podem agravar ao longo do tempo, aumentando as chances de morbimortalidade desses pacientes.

**Conclusáo:**

a triagem dos pacientes e dos profissionais da saúde para COVID-19 sao estratégias importantes para evitar a contaminacao desses sujeitos. A suspensao e o adiamento de cirurgias eletivas, durante as ondas de contágio por COVID-19, sao recomendados para aumentar a capacidade de leitos disponiveis para pacientes graves hospitalizados por essa doenca. Essa recomendacao também auxilia no remanejamento de profissionais desse setor para as unidades com a demanda de cuidados de saúde mais elevada.

## Abstract (en)

**Introduction:**

Health systems were challenged to develop organizational strategies for the delivery of surgical care.

**Objective:**

to present the strategies of health services with regard to surgical care practices in times of the COVID-19 pandemic.

**Materials and methods:**

An integrative review was conducted following six stages; the search for articles was performed using the Virtual Health Library, SciELO, PubMed, and ScienceDirect. The science descriptors adopted were “Surgicenters” and “Coronavirus Infections,” according to the DeCS-MeSH terms system. We selected 60 articles from 405 studies found.

**Results:**

The main strategies used by the health services were to suspend and postpone elective surgeries during waves of disease transmission and careful screening of patients with COVID-19 before and after surgery.

**Discussion:**

The suspension and postponement of elective surgeries should be carefully evaluated by the health team, individually for each patient since non-urgent clinical situations may worsen over time, increasing the chances of morbidity and mortality in these patients.

**Conclusion:**

screening patients and health professionals for COVID-19, according to the reality of surgical units, centers or outpatient clinics, is an important strategy to avoid contamination of these subjects. The suspension and postponement of elective surgeries, during the waves of contagion by COVID-19, is recommended to increase the capacity of beds available for critically ill patients hospitalized for this disease. This recommendation also helps in the reassignment of professionals in this sector to the units with the highest demand for health care.

## Resumen

**Introducción:**

los sistemas de salud fueron desafiados a desarrollar estrategias organizacionales para la prestación de atención quirúrgica.

**Objetivo:**

presentar las estrategias de los servicios de salud en relación a las prácticas de atención quirúrgica en tiempos de la pandemia por COVID-19.

**Materiales y métodos:**

Revisión integradora, desarrollada en seis etapas, cuya búsqueda de artículos se realizó en la Biblioteca Virtual en Salud, SciELO, PubMed y ScienceDirect. Los descriptores de salud adoptados fueron “Centros Quirúrgicos” e “Infecciones por Coronavirus”, según los sistemas DeCS y MeSH Terms. Seleccionamos 60 artículos de 405 estudios encontrados.

**Resultados:**

Las principales estrategias utilizadas por los servicios de salud fueron: la suspensión y postergación de cirugías electivas durante las oleadas de contagio de la enfermedad; y la detección cuidadosa de pacientes para COVID-19 antes y después de las intervenciones quirúrgicas.

**Discusión:**

La suspensión y postergación de cirugías electivas debe ser cuidadosamente evaluada por el equipo de salud, ya que situaciones clínicas no urgentes pueden empeorar con el tiempo, aumentando las posibilidades de morbilidad y mortalidad en esos pacientes.

**Conclusión:**

El triaje de dos pacientes y dos profesionales de la salud para COVID-19 son estrategias importantes para evitar la contaminación de estos sujetos. Se recomienda la suspensión y postergación de cirugías electivas, durante las olas de contagio por COVID-19, para aumentar la capacidad de camas disponibles para pacientes críticos hospitalizados por esta enfermedad. Esta recomendación también ayuda en la reasignación de profesionales de este sector a las unidades con mayor demanda de atención en salud.

## Introdujo

A pandemia causada pela COVID-19 afetou as práticas de cuidados cirúrgicas em todo o mundo^1^. Em vista disso, os profissionais da saúde tém visto a necessidade de que sejam formuladas e discutidas estratégias para manter a realizado dos procedimentos cirúrgicos sem prejuízo as equipes e aos pacientes^2^.

O novo coronavírus (SARS-CoV-2) pode se apresentar sob a forma de aerossóis e se manter sob superficies, disseminando-se principalmente através de gotículas em suspensao e por fómites. Esse agente infeccioso pode permanecer infectante por horas no ar, superficies plásticas, ago inoxidável, papelao e cobre. O risco de contaminagao por esse microrganismo é elevado, especialmente nos ambientes de cuidados cirúrgicos, pois diversos procedimentos realizados nesses locais sao passíveis de formagao de gotículas e aerossóis^3^.

O cenário global vivenciado no ano de 2021 atesta novas ondas de contaminagao do SARS-CoV-2^4^. Por isso, diante desse novo contexto epidemiológico, é necessário reorganizar os servigos de saúde, principalmente no ámbito das práticas de cuidados cirúrgicos, quanto a formulagao de estratégias para a realizagao oportuna e segura do atendimento e do tratamento dos pacientes cirúrgicos. A pandemia de COVID-19 levou a uma superlotagao dos servigos gerais de saúde e, com isso, precisaram diminuir o número de procedimentos eletivos para atender essa nova demanda^1^. Nos centros e ambulatórios cirúrgicos, essa medida visou evitar a disseminagao dessa doenga e prevenir complicagóes pós-operatórias associadas a infecgao pelo novo coronavirus.

Contudo, tais medidas podem causar repercussóes negativas a saúde dos individuos se nao forem devidamente planejadas. Condigóes benignas e malignas, mesmo estabilizadas, podem se tornar incapacitantes, irreversiveis e/ou evoluir para a instabilidade do paciente. Dessa maneira, isso pode ocasionar piora na saúde da populagao, faléncia dos sistemas de saúde, devido ao acúmulo da lista de espera por procedimentos cirúrgicos e custos sociais elevados, visto que os órgaos governamentais precisaram custear aumentos substanciais no volume cirúrgico para solucionar as pendéncias^5^^-^^6^.

Em adigao, os sistemas de saúde de vários paises estao sendo desafiados a desenvolver estratégias organizacionais para manter a prestagao de cuidados cirúrgicos aos pacientes. A vista disso, as instituigóes e profissionais de saúde devem estar aptos para atender a essa demanda, pois o risco de novos colapsos nos sistemas de saúde é real, frente as novas ondas de contaminagao e a coexisténcia de tratamentos atrasados^7^.

Portanto, os riscos de expor os pacientes a infecgao perioperatória pelo SARS-CoV-2, durante o percurso da realizagao dos procedimentos cirúrgicos, sao altos, mas devem ser analisados contra os riscos de atrasos prolongados no tratamento^8^. Dessa forma, faz-se necessário formular e divulgar as estratégias produzidas pelas equipes de saúde dos centros cirúrgicos, frente a realizagao segura de cirurgias.

Destarte, ressalta-se a importáncia de apresentar o conteúdo produzido na literatura acerca das estratégias organizacionais utilizadas para manter a seguranga do paciente e da equipe no periodo perioperatório, de forma que os procedimentos possam ser ofertados sem prejuizo na espera, mantendo a adesao aos protocolos e diretrizes para cirurgias seguras. Além disso, o conhecimento apresentado através deste estudo pode auxiliar as instituigóes e equipes de saúde de centros cirúrgicos a formularem suas estratégias para a manutengao da realizagao de suas práticas de cuidados cirúrgicos, prestando uma assisténcia em saúde multidisciplinar segura.

Nesse contexto, elencou-se a seguinte questao norteadora: quais as estratégias tragadas pelos servigos de saúde de cuidados cirúrgicos, para a manutengao do seu funcionamento seguro, durante a pandemia de COVID-19? Assim, o objetivo deste estudo foi apresentar o conhecimento produzido na literatura científica sobre as estratégias dos servidos de saúde no que se refere as práticas de cuidados cirúrgicos em tempos de pandemia de COVID-19.

## Materiais e Métodos

Trata-se de uma revisáo da literatura do tipo integrativa, caracterizada como um método de investigado que possibilita reunir, analisar e sintetizar pesquisas disponíveis sobre determinados temas de forma sistematizada. Para a elaborado desta revisao integrativa, seguiram-se as seguintes etapas^9^:

1) identificado do tema e construgáo da questáo norteadora da pesquisa; 2) identificado do problema e objetivo do estudo; 3) busca de literatura; 4) coleta dos dados; 5) análise crítica dos resultados; e 6) apresentado da síntese.

O tema de interesse foi a organizado dos servidos de saúde no que se refere as práticas de cuidados cirúrgicos seguros em tempos de pandemia de COVID-19. Nesses termos, formulou-se a seguinte questáo norteadora: quais as estratégias tragadas pelos servigos de saúde de cuidados cirúrgicos para a manutengáo do seu funcionamento seguro durante a pandemia de COVID-19?

A etapa de coleta de dados foi realizada a partir de consultas em bibliotecas, bases de dados e buscador académico: Biblioteca Virtual de Saúde (BVS), Scientific Electronic Library Online (SciELO), National Library of Medicine (PubMed) e Science Direct. As bases de dados acessadas, a partir da BVS, foram: Banco de Dados de Enfermagem (BDENF), Literatura Latino-Americana e do Caribe em Ciéncias da Saúde (LILACS), Sistema Online de Busca e Análise de Literatura Médica (MEDLINE), Índice Bibliográfico Español en Ciencias de la Salud (IBECS), Coleciona SUS, Base Internacional de Guias GRADE (BIGG) e Bibliografía Nacional en Ciencias de la Salud Argentina (BINACIS).

Para as bases de dados acessadas a partir da BVS, adotaram-se os Descritores em Ciéncias da Saúde (DeCS), os quais foram cruzados da seguinte maneira: "Centros Cirúrgicos" AND "Infecgóes por Coronavirus". Para as buscas internacionais, adotaram-se os descritores controlados pelo Medical Subject Headings of U.S. National Library of Medicine (MeSH), realizando-se os seguintes cruzamentos: "Surgicenters" AND "Coronavirus Infections". O cruzamento entre os descritores com o operador booleano "AND" foi necessário, a fim de identificar apenas artigos que apresentassem os dois termos no mesmo estudo.

Definiram-se como critérios de inclusáo os artigos científicos publicados e disponíveis eletronicamente na íntegra em portugués, inglés e espanhol, entre janeiro de 2020 e maio de 2021. O acesso aos estudos que náo estavam disponíveis na íntegra nas bases de dados ocorreu a partir da Plataforma CAPES. Os artigos que se repetiram nas bases de dados e que náo responderam a questáo norteadora foram excluídos. A etapa de busca bibliográfica ocorreu entre fevereiro e maio de 2021. As bases de dados foram acessadas por um só pesquisador, em horários diferentes, sendo todas esgotadas em um único dia, com gravagáo da página de busca. A fase de busca e selegáo dos estudos foi pareada.

**Figura 1:**
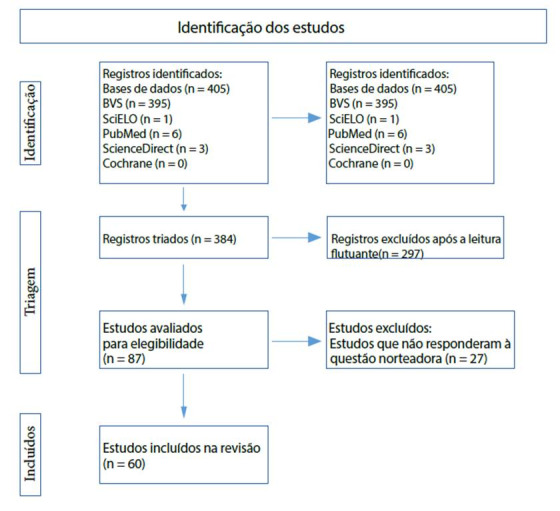
Fluxograma com os resultados da selecáo dos estudos baseado no modelo PRISMA

Adiante, na Figura 1, encontram-se os resultados da busca bibliográfica nas bases de dados, onde está descrita a devida distribuigáo dos artigos encontrados e selecionados. O processo de identificagáo, triagem e inclusáo está descrito em forma de fluxograma, baseado no modelo PRISMA10.

Observa-se que foram encontrados 405 artigos. Todos foram lidos a partir de uma leitura dinámica e flutuante, dada pela apreciado dos títulos e resumos, selecionando-se aqueles que possuíam interface com o tema de estudo para uma leitura na íntegra. Destes, 318 (78,51%) foram excluídos por se tratarem de manuscritos duplicados, nao serem artigos (dissertagóes, teses, manuais e links de vídeos) e/ou por nao responderem a questao norteadora desta revisao. Analisaram-se, exaustivamente, 87 (21,48%) artigos relacionados com o assunto, e, desses, 60 (14,81%) responderam as questóes norteadoras. Os estudos duplicados encontravam-se dentro das próprias bases e dentro da própria BVS.

Um formulário de coleta de dados validado foi utilizado para a coleta dos dados de cada artigo da amostra final, e esse instrumento permitiu a aquisigao de informagóes sobre a identificagao do artigo, tipo de publicagao, características metodológicas do estudo e nivel de evidencia^11^. O conjunto de dados deste estudo foi salvo no repositório público Mendeley Data^12^.

## Resultados

A maioria dos estudos selecionados provém de bases de dados acessadas por meio da BVS (n = 57; 95%). A maioria foi publicado no ano de 2020 (n = 58; 96,66%), e os demais foram publicados no ano de 2021 (n = 2; 3,3%). Os periódicos que mais publicaram sobre a temática pesquisada foram os da área médica (n = 45; 75%). Os estudos estao disponíveis na língua inglesa, espanhola e portuguesa, estando a maioria deles em língua inglesa (n = 56; 93,3%).

Quanto ao nivel de evidencia dos artigos selecionados, observou-se que a maioria dos estudos traz evidencias de nivel 6, ou seja, derivadas de estudos descritivos ou qualitativos (38,3%). 20 (33,3%) artigos sao de nivel 7, por se tratarem de evidencias advindas de autoridades e/ou relatórios de comités de especialistas. Por fim, 15 (25%) artigos sao evidencias provenientes de estudos de coorte e de caso-controle bem delineados, e os outros 2 (3,3%) estudos trazem evidencias de nível 5.

A seguir, na Tabela 1, sao apresentadas as estratégias que os servidos de saúde tragaram, no intuito de manter as práticas de cuidados cirúrgicos, de forma segura, durante a pandemia de COVID-19.

**Tabela 1:** Estratégias trabadas pelos servidos de saúde de cuidados cirúrgicos para o seu funcionamento seguro durante a pandemia de COVID-19

Estratégias de saúde dos servigos para o funcionamento das unidades cirúrgicas	Artigos (%)
Suspensao e adiamento de cirurgias eletivas durante os picos da doenga^13^^-^^51^	(n = 39; 65%)
Implantagao da telemedicina para avaliagao/acompanhamento dos pacientes com indicagao cirúrgica e conscientizagao da populagao para se utilizar desta ferramenta^25^^,^^28^^,^^36^^,^^37^^,^^39^^,^^43^^,^^44^^,^^52^^-^^57^	(n = 13; 21,6%)
Desenvolvimento de protocolos para reinício e ampliagao gradual do quantitativo de cirurgias eletivas realizadas na fase pós-pico^21^^,^^31^^-^^32^^,^^53^^,^^55^^,^^58^^-^^60^	(n = 8; 13,3%)
Oferta de treinamento constante a equipe cirúrgica: para realizagao segura de cirurgias de urgencia por complicagao ou nao de COVID-19; para o reconhecimentode necessidades de intervengóes intensivas; e para a utilizagao adequada dos Equipamentos de Protegao Individual^27^^,^^29^^,^^36^^,^^40^^,^^61^^-^^65^	(n = 8; 13,3%)
Adaptagao e modificagao cultural, estrutural, organizacional e das práticas do servigo de saúde, para viabilizar o tratamento cirúrgico do paciente^21^^,^^24^^,^^26^^,^^27^^,^^32^^,^^53^^,^^56^^,^^63^^,^^66^^,^^67^	(n = 10; 16,6%)
Realizagao de reunióes online de planejamento multidisciplinar, para discussao sobre pacientes com condigóes cirúrgicas urgentes^46^^,^^53^^,^^57^^,^^68^	(n = 4; 6,66%)
Triagem cuidadosa dos pacientes e dos profissionais de saúde para COVID-19 antes e após uma intervengao cirúrgica^21^^,^^22^^,^^24^^-^^28^^,^^31^^-^^33^^,^^35^^,^^37^^,^^40^^,^^42^^,^^45^^,^^46^^,^^48^^,^^53^^,^^55^^,^^56^^,^^66^^,^^69^^-^^73^	(n = 26; 43,3%)
Estratégias para prevengao de infegao e seguranga de pacientes e profissionais na sala de cirurgia ^21^^,^^22^^,^^24^^,^^27^^,^^31^^,^^41^^,^^44^^,^^48^^,^^54^^,^^57^^,^^64^^,^^66^^,^^69^^,^^70^^,^^72^	(n = 15; 25%)
Estabelecimento de alta precoce para os pacientes internados no pós-operatório, com monitoramento/rastreamento para COVID-19^44^^,^^69^	(n = 2; 3,3%)
Disposigao e adaptagao de checklists para supervisao e norteamento de atividades seguras no servigo cirúrgico^55^^,^^63^^,^^70^	(n = 3; 5%)
Investimento em tecnologias, como impressao 3D, para impressao de matérias e Equipamentos de Protegao Individual e para monitoramento das atividades cirúrgicas^30^^,^^73^	(n = 2; 3,3%)
Manutengao das atividades de educagao dos residentes de saúde com readequagóes^21^^,^^56^	(n = 2; 3,3%)
Desenvolvimento de ferramentas de apoio as decisóes clínicas, para a admissao e monitoramento de pacientes cirúrgicos com ou sem COVID-19^23^^,^^25^^,^^27^^,^^31^^,^^32^^,^^43^^,^^66^^,^^74^	(n = 8; 13,3%)
Desenvolvimento de escalas de trabalho mais flexíveis no servigo cirúrgico^56^^,^^75^	(n = 2; 3,3%)
Formagao de comites multidisciplinares de governanga^27^^,^^36^	(n = 2; 3,3%)
Criagao de unidades cirúrgicas independentes para pacientes que requerem internagao^31^	(n = 1; 1,6%)
Implementagao de equipes de resposta rápida, para transferencias de pacientes e desinfecgao de ambientes que receberam pacientes cirúrgicos com urgencia^33^^,^^63^	(n = 2; 3,3%)
Manter atividades cirúrgicas especializadas, segundo o risco real de mortalidade^34^^-^^35^^,^^46^^-^^47^^,^^57^^,^^67^^,^^74^	(n = 7; 11,6%)
Iniciativas de apoio psicossocial aos profissionais de saúde envolvidos no tratamento de pacientes com COVID-19^36^^,^^63^	(n = 2; 3,3%)
Proibigao da presenga de voluntários ou estagiários para fins educativos e formativos no hospital^46^	(n = 1; 1,6%)

Aproximadamente 98% (n = 54) dos artigos selecionados destacaram, pelo menos, uma informado categorizada como uma estratégia tragada pelos servigos de saúde cirúrgicos, independentemente de serem públicos ou privados, para o atendimento seguro dos pacientes que necessitaram ou que necessitam desse tipo de servigo em meio a pandemia de COVID-19. As estratégias mais citadas e recomendadas foram a suspensao, o adiamento de cirurgias eletivas durante os picos da doenga, para aumentar a capacidade de leitos disponíveis, e a triagem cuidadosa dos pacientes para COVID-19 antes e após sua submissao a procedimentos cirúrgicos. Esses estudos nao revelaram as estratégias que tenham sido implementadas e avaliadas como nao adequadas para a manutengao das práticas de cuidados cirúrgicos.

## Discussao

Diante dos resultados obtidos, as evidencias dos estudos^13^^-^^18^^,^^24^^,^^26^^,^^29^^-^^32^^,^^35^^-^^40^^,^^42^^-^^45^^,^^48^^-^^53^^,^^56^^,^^58^^-^^62^^,^^66^^-^^68^^,^^71^ enfatizam que as estratégias mais utilizadas e recomendadas em diversos hospitais e/ou centros ambulatoriais de referencia cirúrgica nas mais diversas especialidades médicas sao a suspensao e o adiamento de cirurgias eletivas durante os picos da doenga, para aumentar a capacidade de leitos disponíveis. Outros estudos também afirmam que os reagendamentos das cirurgias para um momento oportuno seja o mais ideal, já que a necessidade de equipamentos, equipe e leitos assume um papel relevante, sendo o crescimento da doenga exponencial^73^. Apesar desses achados, ressalta-se que o mais adequado seja analisar caso a caso, para que a demora no atendimento cirúrgico oportuno nao traga prejuízos maiores a condigao clínica do paciente.

Conforme a Agencia Nacional de Vigilancia Sanitária (ANVISA)^74^, essas estratégias de precaugao sao realizadas com o intuito de diminuir a disseminagao da doenga, para que a exposigao desnecessária seja evitada, requerendo das redes hospitalares públicas ou privadas prioridade na assistencia a casos graves da COVID-19. A ANVISA solicitou, ainda, a organizagao de recursos e espagos das instituigóes de saúde, para atender pacientes acometidos pela COVID-19, pois pacientes com COVID-19 podem apresentam maior morbimortalidade no período pós-operatório, sendo que a sua maioria necessita de tratamento em Unidade de Terapia Intensiva (UTI).

Sendo assim, as pesquisas analisadas^19^^,^^20^^,^^23^^,^^28^^-^^30^^,^^32^^-^^38^^,^^42^^-^^44^^,^^47^^,^^48^^,^^50^^,^^53^^,^^56^^,^^60^^,^^61^^,^^64^^,^^66^^,^^70^ salientam que a triagem cuidadosa dos pacientes para COVID-19 antes e após intervengóes cirúrgicas e dos profissionais de saúde sao recomendas. Essa medida possibilita o adequado manejo dos pacientes com suspeita ou confirmagao de infecgao por coronavírus dentro das unidades hospitalares. As recomendagóes que tem sido descritas em outros estudos^75^ nao se contrapóem a esse achado, tendo em vista a necessidade de esses servigos de saúde seguirem diretrizes que tragam condutas eficientes na diminuigao da propagagao do vírus e contaminagao durante os atendimentos. Embora a triagem prolongue o tempo de atendimento do paciente cirúrgico, essa recomendagao é expressamente descrita como sendo recomendada e necessária.

Outras barreiras que influenciam diretamente os servigos de saúde e que foram citadas como estratégias sao as prevengóes de infegao e seguranga do paciente e dos profissionais de saúde. Essa medida é preconizada pelo Ministério da Saúde/ANVISA na Resolugao RDC n° 36 de 25 de julho de 2017^4^, reforgando esse compromisso com a assistencia aos pacientes em diversos ambitos da saúde. A respeito desse compromisso, foi descrito por grande parte dos estudos^20^^-^^21^^,^^23^^,^^29^^-^^30^^,^^32^^,^^37^^,^^42^^,^^47^^,^^54^^,^^57^^,^^59^^,^^64^^,^^66^^,^^72^^)^ que uma das estratégias mais recomendadas dentro das unidades cirúrgicas dizia respeito ao isolamento de casos suspeitos ou confirmados, separagao de salas, ou colocagao de pacientes emsala com pressáo negativa, bem como desinfecto rigorosa de equipamentos e materiais, além do uso de Equipamentos de Protegáo Individual. Esses tópicos listados dizem respeito a estratégia de prevengáo de infecto dentro das unidades cirúrgicas^76^.

No contexto da pandemia de COVID-19, a tecnologia, de fato, veio contribuir ainda mais na assisténcia prestada aos pacientes, sendo o telefone cada vez mais utilizado como um instrumento útil no apoio de prevengáo, diagnóstico e tratamento, ao reduzir o tempo das consultas, evitar o deslocamento de usuários e profissionais e favorecer o acesso a profissionais que desempenham servidos especializados^77^.

Em um estudo publicado sobre a resposta de um servido de cirurgia plástica a COVID-19 em um dos maiores hospitais de ensino da Europa, foi visto que a telemedicina reduziu o contato físico com o paciente, diminuindo o risco de infecto e prorrogando a internato, e, após a alta, continuava- se com o atendimento online . Estando em concordancia com os resultados encontrados pelos autores^13^^,^^19^^,^^21^^,^^33^^,^^34^^,^^35^^,^^38^^,^^49^^,^^50^^,^^52^^,^^58^^,^^59^^,^^72^,foi definida como estratégia o uso da telemedicina para avaliar as indicates cirúrgicas e conscientizagáo da populagáo. No entanto, os custos sáo elevados para se montar uma estrutura de telemedicina, muito embora facilitem os atendimentos e aproximem a populagáo das tecnologias mais modernas^79^.

Sabe-se que toda cirurgia, independentemente do seu porte, precisa, para sua realizagáo, de uma logística mínima, organizagáo, estabelecimento de práticas e implementagáo de novos protocolos e rotinas. Assim sendo, estudos apontam que a adaptagáo e a modificagáo cultural, estrutural, organizacional e de práticas sáo importantes estratégias adotadas e recomendadas durante a

Em concordancia com esses achados, os pesquisadores retratam que é de extrema importancia considerar os riscos, bem como elaborar planos de medidas logísticas para o manejo do paciente cirúrgico suspeito ou confirmado com COVID-19, além de orientar a equipe como melhor atender o paciente^76^.

Os resultados de boa parte dos estudos incluídos nesta revisáo^14^^,^^18^^,^^19^^,^^22^^,^^29^^,^^33^^,^^42^^,^^43^^,^^39^^,^^41^^,^^49^^,^^53^^,^^55^^,^^57^^,^^63^ corroboraram com a compreensáo de que o desenvolvimento de ferramentas de apoio as decisóes clínicas para admissáo, monitoramento, oferta de treinamento para as equipes, intervengóes intensivas, utilizagáo de Equipamento de Protegáo Individual, bem como o desenvolvimento de protocolos antes, durante e após o procedimento cirúrgico e protocolos adotados para reinicio de cirurgias pós-pico de COVID-19, sáo estratégias utilizadas e recomendadas pelos servigos de saúde para funcionamento do centro cirúrgico no momento de pandemia.

A esse respeito, evidencia-se que a adogáo de protocolos torna a comunicagáo e a assisténcia eficazes, proporcionando um cuidado mais seguro e que só se garantem condigóes quando o processo de trabalho é monitorado^77^. Além disso, é essencial a participagáo ativa dos profissionais de saúde para a construgáo efetiva das estratégias.

De acordo com o que foi observado, as instituigóes de saúde devem avaliar epidemiologicamente o local e a regiáo, para considerar a retomada das atividades cirúrgicas, a fim de náo aumentar a possibilidades de contágio entre pacientes e profissionais, levando em consideragáo as taxas de ocupagáo de leitos de UTI e utilizagáo de drogas anestésicas. Portanto, deve-se priorizar as cirurgias de emergéncia, urgéncia e urgéncia eletiva, com especificidade para COVID-19. Para as cirurgias eletivas essenciais e náo essenciais, deve-se considerar o reagendamento^5^. Em consonancia a este estudo, estudiosos^45^^,^^46^^,^^48^^,^^61^^,^^62^^,^^65^^,^^72^ apontam que as atividades cirúrgicas especializadas com risco real de mortalidade específica deveriam ser mantidas pelas instituigóes hospitalares. Em outros estudos, foi discutido o planejamento das cirurgias eletivas mais urgentes, de tal modo que a ideia acima denota a necessidade de avaliar e planejar com base nos riscos de necessidades cirúrgicas^19^^,^^27^^,^^61^^,^^72^.

Observa-se, portanto, que a manutengao das atividades voltadas para educagao dos profissionais nesse período foi fundamental para a implantagao de protocolos de checklist, que norteavam as equipes na realizagao de atividades, rastreamento e monitoramento dos pacientes suspeito ou confirmados. Além de alta precoce, enfatiza-se o uso de tecnologia de ponta, rodízio de escalas, redugao de profissionais para minimizar a exposigao, comités de gestao e uma rede de apoio psicossocial para os profissionais lidarem com a situagao^20^^,^^25^^,^^40^.

Para a organizagao do centro cirúrgico no contexto da pandemia^80^, agóes relevantes necessitam de decisóes compartilhadas entre todos os profissionais, pois oportunizam possibilidades de melhorias e reorganizagao do servigo do centro cirúrgico, garantindo seguranga aos pacientes e a equipe multiprofissional durante a pandemia de COVID-19.

Outros achados relevantes, porém, com pouca citagao^61^, indicavam a proibigao da presenga de voluntários ou estagiários, para fins educativos e formativos no hospital, como estratégia para minimizar os riscos de contaminagao nos servigos de saúde. A criagao de unidades cirúrgicas independentes para pacientes que requerem internagao também apresentou poucas evidéncias.

Diante de tal revisao integrativa, foi possível identificar diversas práticas e estratégias desenvolvidas dentro das instituigóes de saúde, com o intuito de promover a seguranga do paciente e dos profissionais de saúde no contexto da pandemia. Com relagao as limitagóes deste estudo, destaca-se que, embora se tenha obtido uma amostra significante de artigos científicos, nao foi possível esgotar todas as bases de dados existentes e nem realizar mais de uma estratégia de cruzamento de descritores.

## Conclusao

Diante dos resultados apresentados neste estudo, foi possível identificar as principais estratégias formuladas e utilizadas pelos servigos de saúde, para a manutengao das práticas de cuidados cirúrgicos, durante a pandemia de COVID-19. A triagem dos pacientes e dos profissionais de saúde para COVID-19, conforme a realidade das unidades, centros ou ambulatórios cirúrgicos, é importante estratégia para evitar a contaminagao desses sujeitos. A suspensao e o adiamento de cirurgias eletivas durante as ondas de contágio por COVID-19 sao recomendados para aumentar a capacidade de leitos disponíveis para pacientes graves hospitalizados por essa doenga. Essa recomendagao também auxilia no remanejamento de profissionais desse setor para as unidades com a demanda de cuidados de saúde elevada.

Apesar da suspensao e adiamentos de cirurgias eletivas terem sido elencadas como estratégias para a manutengao das práticas de cuidados cirúrgicos em saúde durante a pandemia, destaca-se que essa situagao deve ser avaliada com cautela pela equipe de saúde, de forma individualizada, para cada paciente, visto que situagóes clínicas nao urgentes podem agravar ao longo do tempo, aumentando a chance de morbimortalidade dos pacientes. Estudos sobre a temática com foco nos impactos e na eficiéncia dessas estratégias adotadas para a manutengao das práticas de cuidados nos setores cirúrgicos devem ser realizados.

Por fim, ressalta-se que as evidéncias deste estudo podem auxiliar aos profissionais de saúde atuantes em centros cirúrgicos na elaboragao e utilizagao de estratégias para a manutengao das práticas cirúrgicas de forma segura. Além disso, podem beneficiar a divulgagao de conhecimento científico acerca das mudanzas já realizadas nos servidos de saúde, frente a COVID-19, e podem servir de norteamento para o delineamento de novas estratégias, considerando as novas ondas de contágio da doenga.
